# The Identification of a Novel Fucosidosis-Associated *FUCA1* Mutation: A Case of a 5-Year-Old Polish Girl with Two Additional Rare Chromosomal Aberrations and Affected DNA Methylation Patterns

**DOI:** 10.3390/genes12010074

**Published:** 2021-01-08

**Authors:** Agnieszka Domin, Tomasz Zabek, Aleksandra Kwiatkowska, Tomasz Szmatola, Anna Deregowska, Anna Lewinska, Artur Mazur, Maciej Wnuk

**Affiliations:** 1 Department of Pediatrics and Pediatric Endocrinology and Diabetes, University of Rzeszow, Aleja Rejtana 16c, 35-959 Rzeszow, Poland; agalasek@mp.pl; 2 National Research Institute of Animal Production, Krakowska 1, 32-083 Balice, Poland; t.zabek@izoo.krakow.pl (T.Z.); tomasz.szmatola@urk.edu.pl (T.S.); 3 Laboratory of Exercise Physiology and Biochemistry, Institute of Physical Culture Studies, College of Medical Sciences, University of Rzeszow, Aleja Rejtana 16c, 35-959 Rzeszow, Poland; akwiatkowska@ur.edu.pl; 4 University Centre of Veterinary Medicine, University of Agriculture in Krakow, Al. Mickiewicza 24/28, 30-059 Krakow, Poland; 5 Department of Biotechnology, University of Rzeszow, Aleja Rejtana 16c, 35-959 Rzeszow, Poland; deregowskaanna@o2.pl (A.D.); alewinska@o2.pl (A.L.)

**Keywords:** fucosidosis, *FUCA1* mutation, rare chromosomal aberrations, 15q11.1-11-2 microdeletion, Xq22.2 gain, DNA methylation

## Abstract

Fucosidosis is a rare neurodegenerative autosomal recessive disorder, which manifests as progressive neurological and psychomotor deterioration, growth retardation, skin and skeletal abnormalities, intellectual disability and coarsening of facial features. It is caused by biallelic mutations in *FUCA1* encoding the α-L-fucosidase enzyme, which in turn is responsible for degradation of fucose-containing glycoproteins and glycolipids. *FUCA1* mutations lead to severe reduction or even loss of α-L-fucosidase enzyme activity. This results in incomplete breakdown of fucose-containing compounds leading to their deposition in different tissues and, consequently, disease progression. To date, 36 pathogenic variants in *FUCA1* associated with fucosidosis have been documented. Among these are three splice site variants. Here, we report a novel fucosidosis-related 9-base-pair deletion (NG_013346.1:g.10233_10241delACAGGTAAG) affecting the exon 3/intron 3 junction within a *FUCA1* sequence. This novel pathogenic variant was identified in a five-year-old Polish girl with a well-defined pattern of fucosidosis symptoms. Since it is postulated that other genetic, nongenetic or environmental factors can also contribute to fucosidosis pathogenesis, we performed further analysis and found two rare de novo chromosomal aberrations in the girl’s genome involving a 15q11.1-11.2 microdeletion and an Xq22.2 gain. These abnormalities were associated with genome-wide changes in DNA methylation status in the epigenome of blood cells.

## 1. Introduction

Fucosidosis (OMIM# 230000) is a very rare neurodegenerative disorder, classified as a lysosomal storage disease (LSD). To date, approximately 120 cases have been reported, mostly in Italy, Cuba and the USA [1]. Fucosidosis is inherited as an autosomal recessive trait and is caused by deficiency in α-L-fucosidase (EC 3.2.1.51)—an enzyme engaged in the degradation of fucose-containing glycoproteins and glycolipids [2]. The α-L-fucosidase is encoded by *FUCA1* (α-L-Fucosidase 1), which is localized on chromosome 1p36.11 and consists of eight exons [3]. Both homozygous mutations (when two alleles are mutated at the same locations) and heterozygous mutations (when two alleles are mutated but at different sites) in the *FUCA1* locus can result in a decrease or loss of α-L-fucosidase activity [1,4,5,6]. This in turn impairs the degradation of fucose-containing compounds leading to their deposition in different tissues. Consequently, progressive accumulation of the fucosylated substrates aggravates the clinical course of fucosidosis [1,4,5,6]. Although the clinical manifestation and severity of fucosidosis is broad, it largely involves neurodegeneration with progressive mental, motor and neurological deterioration, as well as growth retardation and coarse facial features [1,4,6].

To date, 36 pathogenic variants in the *FUCA1* gene have been shown to cause deficiencies of α-L-fucosidase and, thereby, to be linked to fucosidosis. Among them, only three splice site variants have been documented and the identified substitutions affected: (i) the invariant GT donor splice site of intron 4 (nucleotide change: c.768+1G>A; Human Gene Mutation Database (HGMD) accession: CS1711597) [1,7,8]; (ii) the invariant GT donor splice site of intron 5 (nucleotide change: c.969+1G>A; HGMD accession: CS930812) [1,8,9] and (iii) the invariant AG acceptor splice site of intron 7 (nucleotide change: c.1261-1G>A; HGMD accession: CS1913889) [1,8,10].

Here, we report a novel 9-base-pair deletion (NG_013346.1:g.10233_10241delACAGGTAAG) associated with fucosidosis covering the junction between exon 3 (four bases) and intron 3 (five bases) within the *FUCA1* sequence. This homozygous deletion was identified in a five-year-old girl with clinical suspicion of fucosidosis and was associated with a 2.6-fold decrease in α-L-fucosidase activity in her whole blood samples, thereby confirming the diagnosis.

Due to the poor genotype–phenotype correlations, the broad extent of severity and range of clinical variability of fucosidosis, it has been postulated that other genetic, nongenetic and/or environmental factors contribute to its pathogenesis and clinical course [1,11]. For example, the epigenetic regulatory system, including DNA methylation, is a well-known contributor to the broadening of the phenotype [12]. The pattern of the 5-methylcytosine (5mC) across the DNA defines transcriptionally active and inactive chromatin regions, thereby regulating numerous developmental and physiological processes [13]. Also, it is known that disease-related changes in DNA methylation can lead to genetic instability mediated by accelerated telomere shortening [14]. Although we indeed observed genome-wide changes in the DNA methylation pattern in the epigenome of the girl’s blood cells that involved a wide range of the metabolic-related and biochemical pathways, we did not find any changes in telomere length. Instead, we identified two de novo rare chromosomal aberrations in the girl’s genome encompassing a microdeletion of the 11.1-11.2 segment of the long arm of chromosome 15 (15q11.1-11.2) and a gain of the 22.2 region within the long arm of chromosome X (Xq22.2) that together involved four OMIM genes.

In summary, in this study, we identify a novel pathogenic variant in the *FUCA1* gene that is linked to fucosidosis. We also discuss the role of the interplay between genetic and epigenetic factors in the pathogenesis of the disease and its clinical manifestations.

## 2. Materials and Methods

### 2.1. PCR Amplification and Sanger Sequencing of FUCA1 Exons

DNA was isolated from whole blood using a Wizard Genomic DNA Purification Kit (Promega, Poland). All subjects gave their informed consent for inclusion before they participated in the study. The study was conducted in accordance with the Declaration of Helsinki and the protocol was approved by the Ethics Committee of University of Rzeszow (19 June 2018). The sample set included persons from the investigated family trio and the blood of a single unrelated person. The PCR assay encompassed amplification of nine DNA fragments covering exons in the region of the *FUCA1* reference sequence (chr1: 23845347–23867835 from GRCh38 version of the human genome) (Table 1). PCR primers were designed using the Primer3 software [15,16] and synthesized by Genomed S.A. (Warsaw, Poland). Amplification was performed in 30 PCR cycles under an annealing temperature (Ta) specified in Table 1 using hot start Taq DNA polymerase (Qiagen, Hilden, Germany). The thermal program included an initial step of 95 °C for 15 min, 30 cycles of denaturation at 94 °C for 30 s, 1 min of annealing and 1 min of extension at 72 °C and a final extension step at 72 °C for 7 min. In the case of primers designed for the region of exon 5, a touchdown PCR procedure (TDPCR) was applied in the first stage of amplification. The initial TDPCR stage included 11 steps of annealing temperature decreases of 0.5 °C in the range from 65 °C down to 59.5 °C. The second stage of TDPCR included 30 PCR cycles with annealing at 59 °C. PCR amplifications were done on the VeritiPro Thermal Cycler (Thermo Fisher Scientific, Warsaw, Poland). Amplified DNA samples were run in two percent agarose gel stained with ethidium bromide. Products revealing clear PCR bands were subjected to Sanger sequencing using PCR primers and the BigDye™ Terminator v3.1 Cycle Sequencing Kit (Thermo Fisher Scientific, Warsaw, Poland). The manufacturer-recommended cycle sequencing program was applied with the primer annealing of 59 °C and 4 min of extension at 60 °C. Sequencing of PCR products was done on the VeritiPro Thermal Cycler (Thermo Fisher Scientific, Warsaw, Poland). Sequencing products were cleaned with the BigDye XTerminator™ Purification Kit (Thermo Fisher Scientific, Warsaw, Poland) and electrophoresed on the ABI3500xl genetic analyzer (Thermo Fisher Scientific, Warsaw, Poland).

Obtained sequencing traces were evaluated for quality and subjected to variant genotyping using FinchTV 1.4.0 (Geospiza Inc., Seattle, WA, USA) and Variant Analysis (VA) software (Applied Biosystems, Foster City, CA, USA). Further investigations included trace sequence alignment against reference sequences using GENEDOC software [17] to check for the variation among investigated DNA samples. Obtained sequence traces were also aligned against a reference sequence of the human genome using the blastx option of the NCBI website in order to check for their homology to the translated sequences encoding FUCA1 protein isoforms. GENEDOC software was used for DNA sequence translation of reported DNA variants.

### 2.2. Array-Based Comparative Genomic Hybridization

The genome-wide array-based comparative genomic hybridization (aCGH) was performed using SurePrint G3 Human CGH 60 k Oligo Microarrays (Agilent Technologies, Santa Clara, CA, USA). CGH analysis was performed according to the manufacturer’s protocol (Agilent Technologies). The array slides were scanned (SureScan Microarray C scanner, Agilent) and analyzed using CytoGenomic Workbench software (Agilent). The procedure was conducted by Genomed S.A. (Warsaw, Poland).

### 2.3. Reduced Representation Bisulfite Sequencing

The chemistry of Premium Reduced Representation Bisulfite Sequencing (RRBS) Kit (Diagenode, Sraing, Belgium) was used for preparation of RRBS libraries. The 100 ng of prepared DNA were digested with methylation insensitive restriction enzyme MspI to generate short fragments—each containing at least one CpG site. After end repair, A-tailing and ligation to methylated adapters with separate indexes, the CpG-rich fragments were selected according to size (Agencourt AMPure XP, Beckman Coulter, Poland), subjected to bisulfite conversion and PCR amplified. Reduced representation bisulfite sequencing (RRBS) libraries were pooled in groups of six and sequenced in a 75 bp single-end read on the NextSeq 550 system (Illumina) (Admera Health, La Jolla, CA, USA). Data were deposited in SRA database (SRA accession: PRJNA655811).

### 2.4. Raw Data Analysis

Analyses were performed using the bsmap and methylKit software packages with default settings set for the RRBS mode [18,19]. CpG methylation calling was performed using sequence reads with at least 10x coverage. Next generation sequencing (NGS) reads were first checked for quality (FastQC software, available online: http://www.bioinformatics.babraham.ac.uk/projects/fastqc/ and https://qubeshub.org/resources/fastqc, available online: https://qubeshub.org/resources/fastqc) and filtered using Flexbar software [20]. The filtered reads were aligned to the human reference sequence using Bsmap software with the default setting set for the RRBS mode and 10% of read length considered as mismatches. We set the threshold level of the differential methylation value between samples as equal to or below the value of −25 (the meaning of F vs. D or M vs. D hypomethylation) and equal to or above value of +25 (the meaning of F vs. D or M vs. D hypermethylation) (Supplementary Table S1). We combined the information about the genomic positions of the identified differentially methylated sites (DMS) resulting from the comparison of F vs. D and M vs. D RRBS datasets (Supplementary Table S2). The combined set of F vs. D and M vs. D differentially methylated sites was annotated in Biomart and implemented in the Ensembl database, applying the GRCh38 version of the human genome (Supplementary Table S2).

### 2.5. The Variant Calling Procedure

After mapping, respective Read Groups were added to each sample and duplicates were marked with Picard Tools [21]. Then, Bis-SNP software [22] was used to maintain the variant calling procedure. The filtration of the obtained variants was done with the VCF tools software [23] with the following parameters: minimum coverage set to seven; minimum quality set to 20.

### 2.6. Kyoto Encyclopedia of Genes and Genomes Pathway Description and Functional Annotation of DMS Associated Genes

In order to describe the biological role of the differentially methylated genes, we first used the DAVID annotation tool to retrieve the list of Kyoto Encyclopedia of Genes and Genomes (KEGG) pathways for all differentially methylated genes found in this study (results included in Supplementary Table S5). We also conducted gene enrichment analysis with the use of the DAVID functional annotation tool (false discovery rate below 0.05) [24], separately for genes linked to hypo- and hypermethylated DMS (results included in Supplementary Table S4).

Heatmaps were generated using Genesis 1.8.1 [25].

### 2.7. Telomere Restriction Fragment Length

Mean Telomere restriction fragment (TRF) length was measured by Southern blot analysis using the TeloTAGGG telomere length assay kit (Roche, Warsaw, Poland) according to the manufacturer’s instructions. Mean TRF length was analyzed following Wnuk et al. [26].

## 3. Results

### 3.1. Case Presentation

A three-year-old girl was diagnosed in the Children’s Endocrinology Clinic due to delayed psychomotor development and short stature.

No family history of neurodegenerative disease was reported. Parents were healthy with typical facial features.

The girl was a second child, a sister of a healthy boy, of nongenetically-related parents. She was born after a full-term, uneventful pregnancy. She met her developmental milestones at age-appropriate intervals and the parents also reported her normal growth up to 12 months. After that, an initial stagnation of development started followed by progressive spasticity of the body. At the age of 13 months, she started to walk but for a long time with an uncertain, awkward gait with frequent falls.

She was also examined at the Pediatric Department in the second year of life, where a radiological examination of the cervical spine and chest (no pathology in the field of bone structures, no dysostosis multiplex), electroencephalography (EEG) (basic activity recorded as too slow for her age) and brain magnetic resonance imaging (MRI) (no focal lesions, symmetrical ventricular system, corpus callosum of correct size were found) were performed.

At the age of three, physical examination showed facial dysmorphia, namely coarse facial features, protruding forehead, arcuated eyebrows and broad eye gap. Her lips were thick and macroglossia was found. On the dry skin, a red maculopapular rash (suspected telangiectasia) was found, which extended to the whole body, including palms and soles, with increasing age. No hepatosplenomegaly was noted.

At the time of her assessment, her weight was 16.5 kg (50th–85th percentile) and her height was 97.5 cm (50th percentile) with a BMI of 17.36 kg/m^2^ (85th–97th percentile).

In the neurological examination—conscious, no logical verbal contact—she carried out simple commands supported by gesture, made eye contact, followed the toy with her eyes, her facial expressions when crying were symmetrical, muscle tension was increased, she showed deep reflexes and demonstrated a symmetrical, independent gait walking on the toes with a limp in the right lower limb. In addition, left torticollis and abolition of physiological curvatures of the spine (deepening of thoracic kyphosis and straightening of lordosis in the lumbar region) were found.

Laboratory tests showed no significant deviations except with regard to vitamin D (25 (OH) D3 deficiency: 15.5 ng/mL (N: 20–60 ng/mL)), slightly elevated ammonia (97.0 µg/dL (27.0–90.0)), aspartate aminotransferase (AST, 66 U/L (N: 0.0–35.0)) and alkaline phosphatase (333 U/L (N: 96–297)). Other tests were normal, including with regard to lactic acid, acid-base balance, alanine aminotransferase and uric acid (Supplementary Table S1).

Abdominal ultrasound showed no visceromegaly (including liver or spleen). Ophthalmoscopic examination showed a normal fundus image (no cherry red spots). Echocardiographic examination showed moderate left ventricular wall hypertrophy (up to 8–9 mm). ECG indicated 170/min sinus rhythm, normogram, PQ, QTc, QRS normal.

The diagnostics were expanded towards storage disorders. Urinary mucopolysaccharides were quantified and found to be elevated (27.3 mg/mmol creatinine (N: 5.88–23.0)). Excretion of urine oligosaccharides was found by thin layer chromatography—a very weak heparan sulfate band was observed in the electrophoresis of glycosaminoglycans. Based on the lysosomal enzyme activity study, MPS IIIA, IIIB, IIIC, IIID, GM1 and mannosidosis were excluded. However, it was found that α-fucosidase activity was decreased (17.89 nmol/mg protein/hour (N: 46 +/− 4)) (Supplementary Table S1).

Subsequently, the girl attended further medical consultations in the metabolic diseases clinic and multifaceted medical interventions (cardiological, neurological, orthopedical) and rehabilitation were implemented.

At the age of four years and ten months, she was hospitalized in the Department of Neurological Rehabilitation for Children due to psychomotor regression (disorders of muscle tone and motor, cognitive and speech development). At that time, an abnormal gait pattern, no self-service and no physiological needs were reported. She was a cheerful girl with limited verbal contact, speaking a few words (indistinctly). She underwent a test with the Children’s Development Scale (DSR) that showed a level of development at the stage of a child of about one year and nice months of age. In the neurologopedic examination, impaired speech development and reduced efficiency of the organs of the articulatory apparatus were found. At that time, her weight, height and head circumference were 18.7 kg (50th–85th percentile), 113 cm (50th percentile) and 52 cm (25th–50th percentile), respectively.

At the age of five, there was a further worsening of the neurological condition. The child demonstrated spastic paresis of the upper left limb and lower limbs, horse feet, pronounced deformity of the right foot and type III walking, independent for short distances, mainly carried by the hand. Over the next months, an increase in spasticity of the upper left limb (deterioration of activity and lack of tweezing grip) was observed. Botulinum toxin and orthoses were used in the treatment.

At the age of five years and two months, she was again hospitalized for rehabilitation. The control examination with use of the DSR revealed her level of development to be at the stage of a child of about one year and 11 months of age. During hospitalization, an exercise was implemented to extend and improve concentration of attention and to improve hand–eye coordination. Progress in cognitive development, compared to the previous hospitalization, was noted.

Currently, it is necessary for the girl to use a baby stroller due to paresis of the left upper limb and lower limbs. A paraparetic gait is possible for short distances. Multifaceted rehabilitation is being continued.

### 3.2. Identification of a Novel Fucosidosis-Related Pathogenic Variant in the FUCA1 Gene

**Figure 1:**
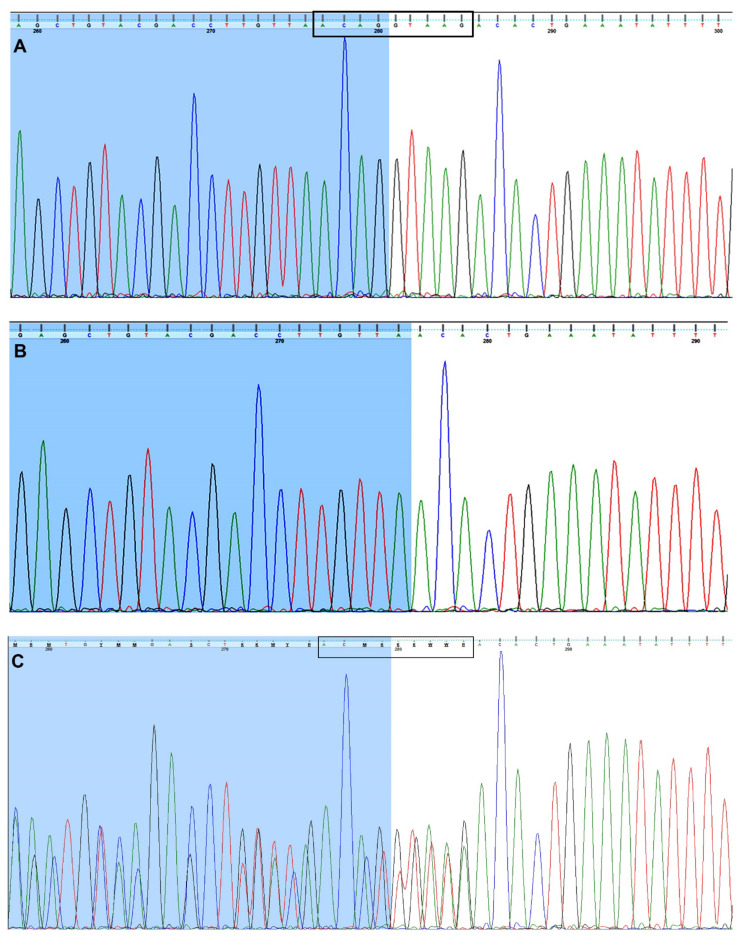
The Sanger-based sequencing. The sequence traces of (**A**) an unrelated person (a control sample), (**B**) the child’s DNA carrying the homozygous 9-base-pair deletion (NG_013346.1:g.10233_10241delACAGGTAAG) encompassing the exon 3/intron 3 junction within the *FUCA1* sequence and (**C**) the girl’s parents. The parents are heterozygous for 9-bp deletion (NG_013346.1:g.[10233_10241delACAGGTAAG]+[=]). (**C**) The bases at the overlapping nucleotide positions are written in the IUPAC code. The sequence of the 3’ end of exon 3 is marked in blue (**A**–**C**).

Sanger sequencing of regions including the *FUCA1* exons revealed a 9-bp deletion (NG_013346.1:g.10233_10241delACAGGTAAG) covering four bases of the sequence at the 3′end of exon 3 and five bases at the beginning of intron 3 in the child diagnosed with metabolic syndrome (MetS) (Figure 1B) compared to control DNA (Figure 1A). Both parents are heterozygous for the mentioned deletion (Figure 1C). The detected deletion was deposited in the ClinVar database under accession number SCV001450462.

**Figure 2:**
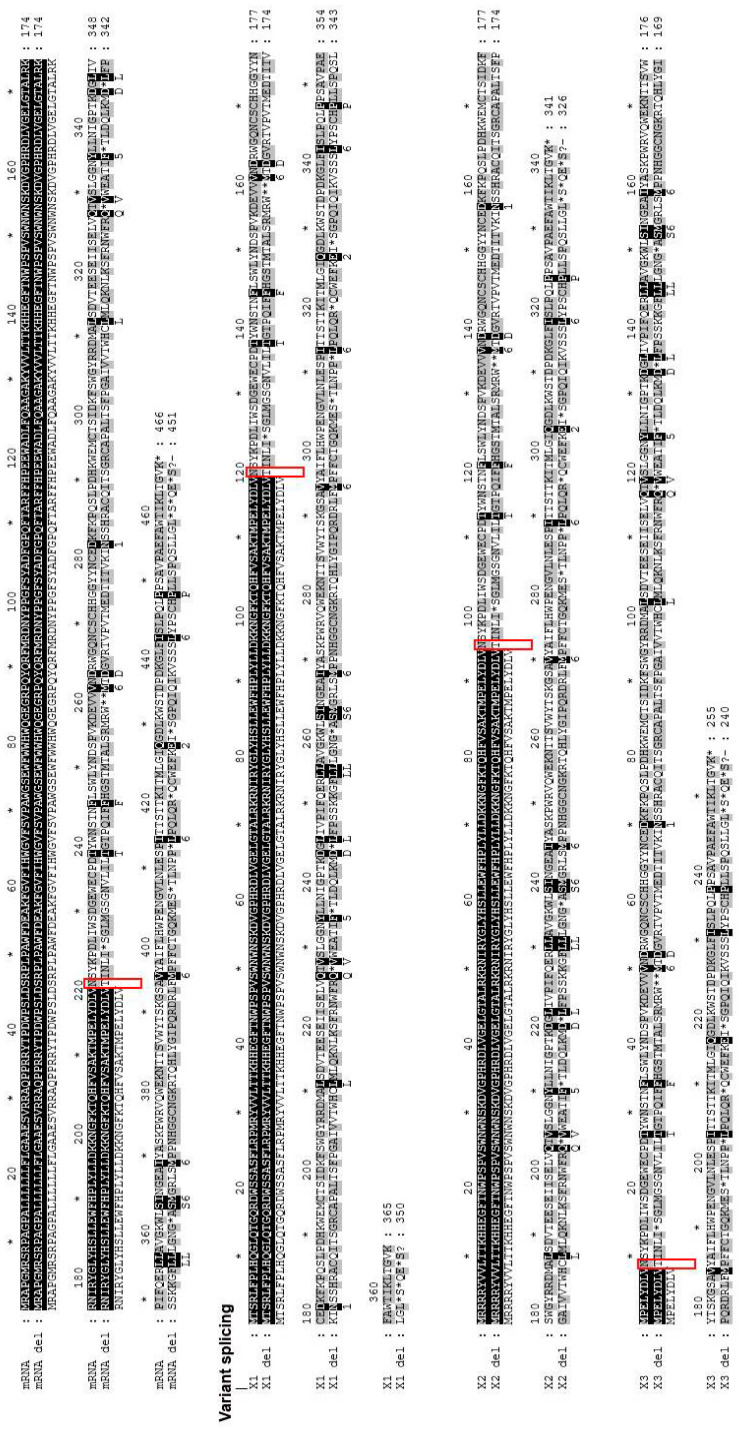
The effect of the 9-base-pair deletion (NG_013346.1:g.10233_10241delACAGGTAAG). The cDNA sequence trace of the child with fucosidosis was translated into amino acids using the blastx option and aligned with both canonical *FUCA1* (NM_000147) mRNA and each of the predicted transcript variants (X1: XM_017000905, X2: XM_005245821 and X3: XM_011541167) translated into corresponding amino acids. Sequence similarity is depicted by different colors, with black indicating the complete similarity of sequences.

This deletion causes frameshift mutation in the *FUCA1* amino acid sequence (N220fs) resulting in the generation of a range of stop codons in encoded proteins (Figure 2). The alignment of the sequence trace of the affected child against human translated cDNA sequences using the blastx option of NCBI showed the disruption of the amino acid sequence of the available *FUCA1* isoforms (data not shown). Further alignment of the *FUCA1* cDNA sequence with the detected deletion (cDNA.663-666delACAG) revealed that the mentioned deletion could be the cause of the frameshift N220fs affecting the main mRNA α-L-fucosidase 1 sequence and all the predicted *FUCA1* isoforms (X1: N120fs, X2: N95fs and X3: N9fs) that could also result in the generation of a range of stop codons in encoded proteins (Figure 2).

Moreover, we found one heterozygous site in the 5 prim UTR region (g. 1:23868297A>G), two at the 3 prim end of exon 1 (g. 1:23868258C>G and g. 1:23868283A>G) and another one located 664 bp downstream of the *FUCA1* gene (g.1:23845053C>G), which are also present in the father of the diagnosed child (data not shown). However, the variants inherited by the child are similar to the reference sequence at the mentioned nucleotide positions.

The obtained RRBS data set allowed us to retrieve genotype data for 529 single nucleotide polymorphism (SNP) markers for which we also checked the inheritance in the investigated family trio (Supplementary Table S2). All of them have known reported variants in the reference SNP database. We also retrieved information about the clinical significance of the mentioned variants. However, comparison of parental and child genotype data with the reference set of alleles showed that the variants present in the child DNA sequence were normal at the loci of the 529 SNP markers.

### 3.3. The Genome-Wide aCGH Analysis Revealed Different Chromosomal Aberrations across the Mother’s and Child’s Genomes

**Figure 3:**
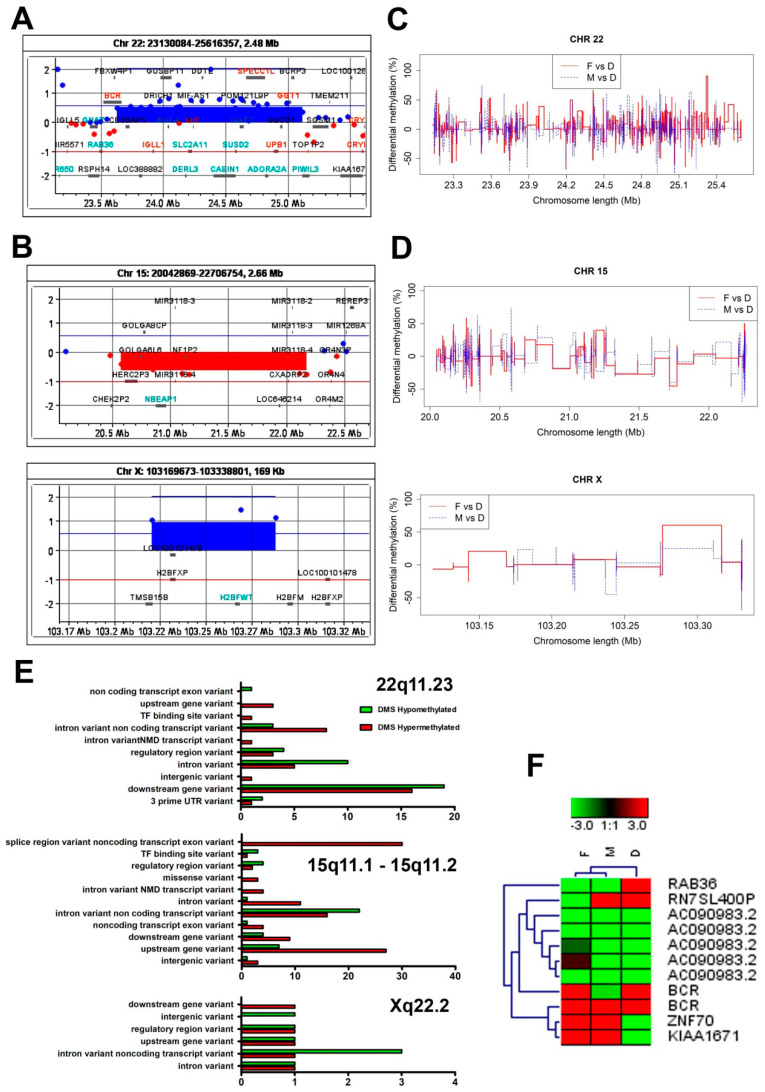
Comparative analysis of the methylation status of chromosomal regions that were subjected to copy number variation in the father’s, mother’s and daughter’s karyotypes. (**A**) An amplification at the q11.23 chromosomal region (a chromosomal gain) on the mother’s chromosome 22 (22q11.23) included the following genes: *BCR*, *CES5AP1*, *ZDHHC8P1*, *LOC101929374*, *LOC388882*, *IGLL1*, *DRICH1*, *GUSBP11*, *RGL4*, *ZNF70*, *VPREB3*, *C22orf15*, *CHCHD10*, *MMP11*, *SMARCB1*, *DERL3*, *SLC2A11*, *MIF-AS1*, *MIF*, *GSTT2B*, *GSTT2*, *DDTL*, *DDT*, *GSTTP1*, *LOC391322*, *GSTT1-AS1*, *GSTT1*, *GSTTP2*, *CABIN1*, *SUSD2*, *GGT5*, *POM121L9P*, *SPECC1L*, *SPECC1L*, *ADORA2A*, *ADORA2A*, *ADORA2A-AS1*, *UPB1*, *GUCD1*, *SNRPD3*, *GGT1*, *LRRC75B*, *BCRP3*, *POM121L10P*, *PIWIL3.* (**B**) A loss at the q11.1-11.2 (a chromosomal microdeletion) region on the daughter’s chromosome 15 (15q11.1-11.2) encompassed the following genes: *HERC2P3*, *GOLGA6L6*, *GOLGA8CP*, *NBEAP1*, *MIR3118-4*, *MIR3118-3*, *MIR3118-2*, *POTEB3*, *POTEB*, *POTEB2*, *NF1P2*, *MIR5701-3*, *MIR5701-1*, *MIR5701-2*, *LINC01193*, *LOC646214*, *CXADRP2*. (**C**) A chromosomal gain at the daughter’s Xq22.2 region included the following genes: *TMSB15B*, *H2BFXP*, *LOC100101478*, *H2BFWT*. (**A**,**B**) The chromosomal amplifications/gains are depicted in blue and the chromosomal losses/microdeletions in red. (**C**,**D**) Differentially methylated sites (DMS) in the pairwise comparisons: father vs. daughter and mother vs. daughter. The comparisons were performed for chromosomes (**C**) 22, (**D**) 15 and X. (**C**,**D**) Pairwise comparisons of DMS values were calculated with the use of methylKit software and then merged and plotted with the use of R software. The pairwise comparisons between F and D and between M and D are depicted in red and blue, respectively. (**E**) The genomic context of the identified DMS at the 22q11.23, 15q11.1-11.2 and X22.2 chromosomal regions. (**F**) A heatmap generated with use of the DMS data obtained from the 22q11.23, 15q11.1-11.2 and X22.2 chromosomal segments. There is an overrepresentation of the DMS within the most divergent group of genes amongst the analyzed chromosomal regions. The heatmap was generated with Genesis 1.8.1. Abbreviations: F—father, M—mother, D—daughter.

Although both the girl and her parents exhibited normal karyotypes in the GTG banding analysis, we considered whether the child’s developmental abnormalities might result from chromosomal microaberrations. We performed a genome-wide aCGH analysis and found that—in contrast to the father’s genome, in which no chromosomal abnormalities were found—both the mother’s and the girl’s genomes exhibited chromosomal aberrations (Figure 3A,B, respectively). We found a gain at the q11.23 segment on chromosome 22 (22q11.23) in the mother’s genome (Figure 3A). In turn, a 15q11.1-q11.2 microdeletion and an Xq22.2 gain (Figure 3B) were confirmed in the daughter’s genome. Since the chromosomal aberrations in the girl’s genome involved different chromosomes than in the mother’s genome, we concluded that the abnormalities in the child’s chromosome structures arose de novo. The list of genes subjected to copy number variations (CNVs) that resulted from the chromosomal aberrations is included in Table 2 and presented in Figure 3A,B.

We also found that several genes that were subjected to copy number variations are critical players in the epigenetic regulatory network and act as linkages for genome-wide epigenetic reprogramming in primordial germ cells (PGCs), oocyte meiosis and genome stability [27,28,29,30]. Since the epigenetic reprogramming in PGCs is strictly linked to DNA methylation, we next asked whether the observed chromosomal aberrations in the child’s genome were associated with changes in DNA methylation patterns.

### 3.4. The Child’s Aberrations of Chromosomes 15 and X Were Associated with Genome-Wide Changes in DNA Methylation Patterns

To answer this question, we performed reduced representation bisulfite sequencing, a technique that combines bisulfite conversion and next generation sequencing and is aimed at enriching genomic regions with a high CpG content [31].

We focused on blood cell methylome differences between the child and her parents and used whole blood samples as material for this analysis. The relevant information, including experiment details of the RRBS and RRBS reads, is available in the SRA database under BioProject PRJNA655811. We employed the RRBS method and obtained between 16 and 24 million NGS reads of high quality, from which 9.8 to 14.4 million were uniquely aligned to the reference sequence of the human genome (GRCh38 version) (more details in Table 3).

The Pearson correlation coefficient of the methylation percentages in all the inspected CpG sites was high between samples, which reflects the global similarities of methylation percentages between samples. All sequenced libraries were characterized by either high or low methylation percentages across the CpG-enriched portion of the genome (Supplementary Figure S1).

Next, we established that there were 10250 differentially methylated CpG sites in the girl’s genome when compared to her parents’ genomes (Supplementary Table S3). In particular, 4048 DMS were hypo- and 6202 were hypermethylated. The analysis of the genome-wide distribution of the DMS revealed that the differentially methylated sites were overrepresented (both hypo- and hypermethylated DMS) within both exonic and intronic sequences in the proximity of coding loci (5 kb up- and downstream of gene boundaries), as well as at regulatory regions. The substantial number of the hypermethylated DMS were located within intergenic regions (Supplementary Figure S2 and Table S4).

We also performed pairwise comparisons of the DMS, i.e., between the father’s (F) and daughter’s (D) DMS as well as between the mother’s (M) and daughter’s (D) DMS. We conducted these analyses separately for hypo- and hypermethylated DMS. All of these data are listed in Table 4 (for details see Supplementary Table S3). The graphical presentations of the pairwise comparisons between F and D, as well as between M and D, DMS with regard to chromosome 22, as well to chromosomes 15 and X, are shown in Figure 1C,D, respectively.

Since chromosomal aberrations were related to the 22q11.23 region in the case of the mother (Figure 3C) as well as to the 15q11.1-11.2 and Xq22.2 segments in the case of the daughter, we conducted a more detailed analysis of these particular chromosomal segments (Figure 3D). We performed a comparative analysis of the methylation status of the 22q11.23, 15q11.1-11.2 and Xq22.2 segments using the reference genome (GRCh38 version) (Figure 3E). This data served as a background for the pairwise comparisons of the DMS analyzed in this study—that is: F vs. D and M vs. D—across the above-mentioned chromosomal regions (Figure 3F). As shown in Figure 3F, hypomethylated DMS encompassed RAB36, while *ZNF70* and *KIAA167*1 were amongst the hypermethylated DMS in the daughter’s genome (compared to both mother’s and father’ genomes) (Figure 3F).

### 3.5. Descriptive Characteristic of Molecular Pathways Relevant for DMS Associated Genes

The Kyoto Encyclopedia of Genes and Genomes analysis revealed that the genes associated with hypomethylated DMS were significantly enriched in the numerous molecular and biochemical pathways related to metabolic processes, cellular signaling, biological processes, neurological processes, cancerogenic processes, physiological processes, cell death and autophagy processes and developmental processes, as well as RNA metabolism (listed in Table 5).

### 3.6. Genes Covering the Differentially Methylated Sites are Involved in a Wide Range of Metabolic and Biochemical Pathways

In order to gain further insight into the biological role of the differentially methylated genes, we conducted a gene set enrichment analysis with the use of the DAVID database. Prior to a functional enrichment analysis, we excluded all loci undergoing parental imprinting to avoid a bias in data interpretation (list available in Supplementary Table S5).

**Figure 4:**
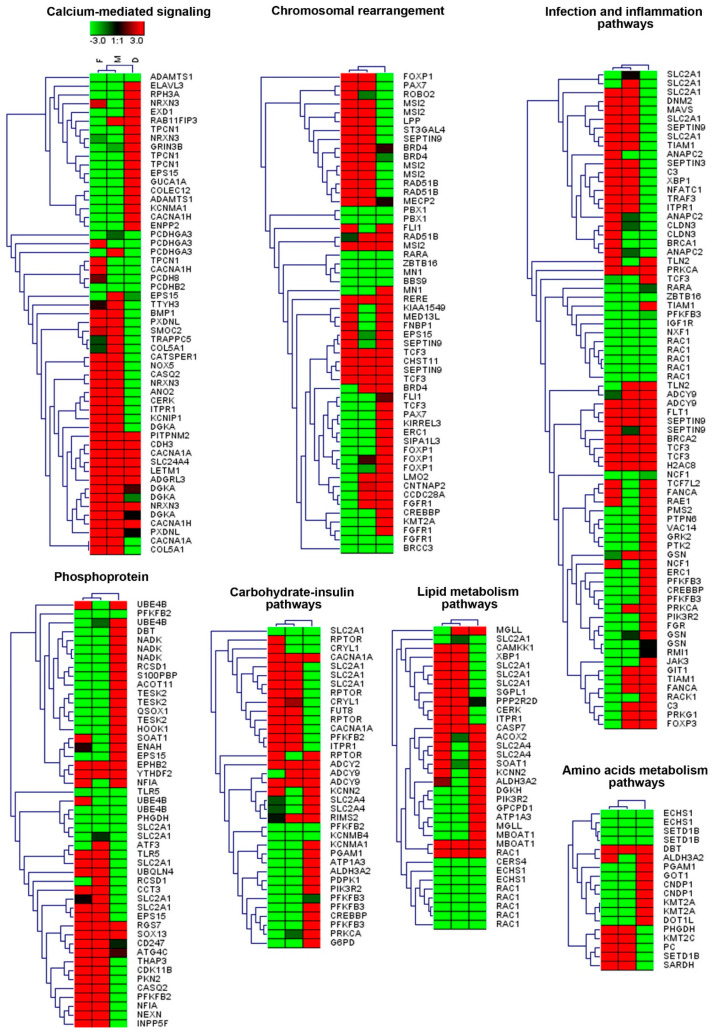
The heatmap analysis of the DMS-associated genes overrepresented for gene ontology (GO) terms and molecular pathways including DMS-specific percentages of methylation for the father (F), mother (M) and daughter (D). Heatmaps were generated using Genesis 1.8.1.

We separately analyzed genes enriched in hypo- and hypermethylated DMS sites and then performed the pairwise comparisons, that is: M vs. D and F vs. D. Gene ontology (GO) analysis, which was performed with the use of the DAVID database, assigned differentially CpG-methylated genes (DMGs) to a range of GO terms. The DMGs were enriched for GO terms related to calcium-mediated signaling, chromosomal rearrangement, infection and inflammation pathways, phosphoprotein, carbohydrate-insulin pathways, lipid metabolism pathways and amino acid metabolism pathways (Figure 4).

The results of the analysis of GO terms with DAVID and a false discovery rate lower than 0.05 (Supplementary Table S6) showed the enrichment of both hypo- and hypermethylated genes in biological processes relevant to cell adhesion and cell junction (GOTERM_BP_DIRECT category). These are mainly genes, which encode the cadherin group of proteins and their repeats (INTERPRO category), as well as phosphoproteins and calcium-related proteins (UP_KEYWORDS category). Among them, a large group included genes encoding 111 phosphoproteins from metabolic pathways and dozens of phosphoproteins from PI3K-Akt, MAPK, cAMP, Rap1, Ras, chemokine, calcium, cGMP-PKG, insulin and thyroid hormone signaling pathways (Supplementary Table S7).

The hypomethylated gene set was overrepresented in the BP processes relevant to transcription and in outflow tract septum development (Supplementary Table S5). These are genes encoding proteins involved in development that also play a role in DNA binding and transcriptional regulation (GOTERM_MF_DIRECT category) (Supplementary Table S5).

A group of exclusively hypermethylated genes were overrepresented in the encoded components of neuromuscular junction (GOTERM_CC_DIRECT) (Supplementary Table S6). Hypermethylation of parental blood samples in relation to the blood of the child with MetS was also associated with overrepresentation of genes encoding the coiled coil and kinase family of proteins as well as proteins involved in chromosomal rearrangement and mental retardation (UP_KEYWORDS category) (Supplementary Table S8). Hypermethylated genes that represented the last two mentioned GO terms were mainly associated with neural conditions, including a variety of types of mental retardation, autism, schizophrenia and epileptic encephalopathy (Supplementary Tables S8 and S9).

### 3.7. Analysis of the Telomere Restriction Fragment Length Showed No Abnormal Telomere Shortening in the Child’s Genome

**Figure 5:**
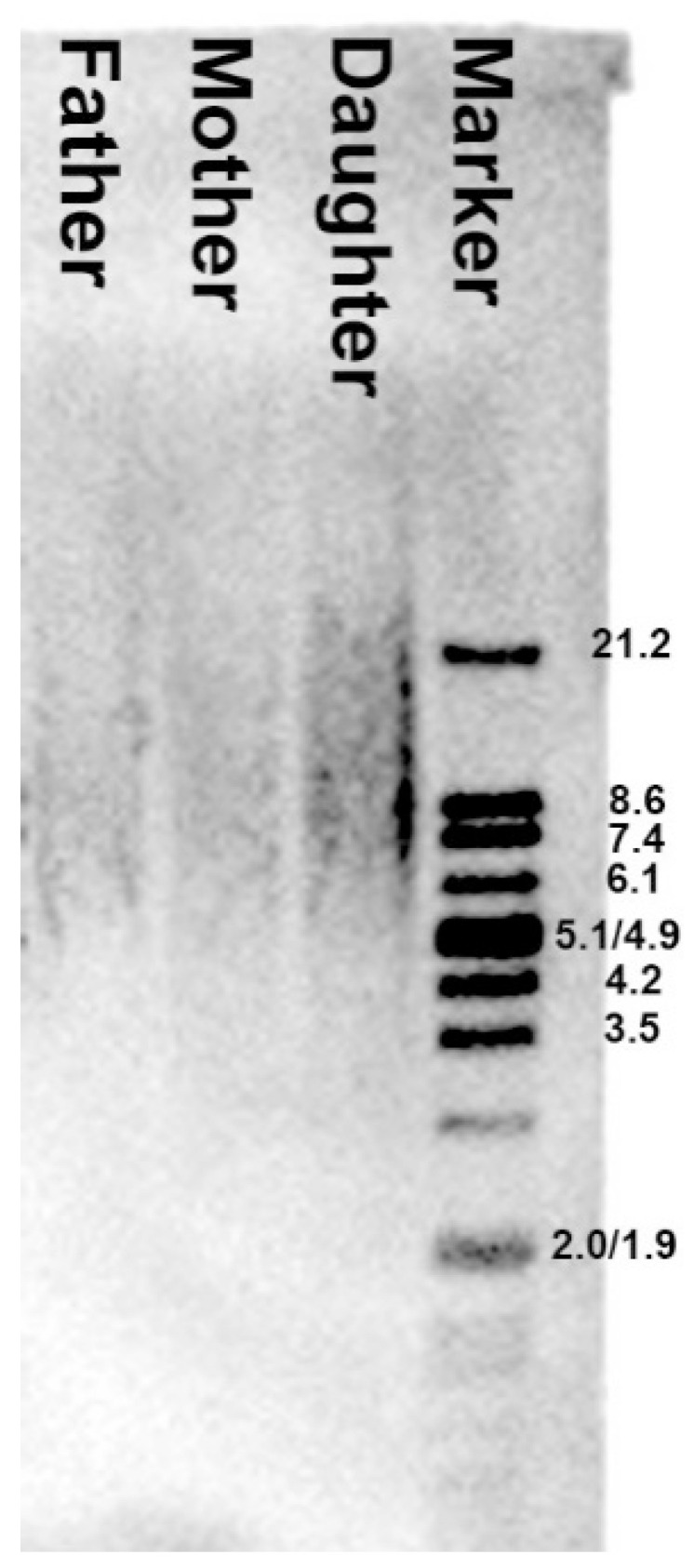
Differences in leukocyte telomere length between parents and child. The mean telomere restriction fragment (TRF) length was measured by Southern blot analysis. Marker—molecular weight marker (kb). The mean TRF length for each sample was provided in kb on the basis of the signal intensity peak from the TRF as a result of multiple hybridization of telomeric-specific hybridization probes. Father ≈ 8.6 kb, mother ≈ 9.0 kb, daughter ≈ 9.3 kb.

It is well known that pathogenic changes in methylation can lead to genetic instability mediated by accelerated telomere shortening [14,32]. However, in this case the TRF analysis did not show any abnormal telomere shortening in the child compared to the parental telomere length (Figure 5). The observed differences in telomere length were related to physiological age rather than pathogenic genomic instability.

## 4. Discussion

In this study we report a novel fucosidosis-related 9-base-pair deletion (NG_013346.1:g.10233_10241delACAGGTAAG) that covers the exon 3/intron 3 junction within the *FUCA1* sequence. This biallelic mutation was identified in a five-year-old Polish girl with clinical manifestation of fucosidosis. Among the 36 documented pathogenic variants in the *FUCA1* sequence, there have been three splice site variants reported to date [1,7,8,9,10]. They are characterized by different single nucleotide substitutions that affect the donor [1,7,8,9] or acceptor [1,8,10] splice sites of different introns.

The c.768+1G>A substitution affecting the invariant GT donor splice site of intron 4 was documented in Syrian patients and was associated with intellectual disability and leukodystrophy. However, no further information concerning the medical or family histories of the patients is available [1,7]. Notably, intellectual disability was also observed in our case. Another substitution, that is, c.969+1G>A, was found to affect the invariant GT donor splice site of intron 5 and was identified in an East Indian–Zambian female [1,8,9]. To some extent, the clinical phenotype of the patient resembled the clinical characteristics that were exhibited by the child reported in this study. The common symptoms included: language skills deterioration, awkward gait with frequent falls, growth retardation, coarse facial features and kyphosis [1,8,9]. In contrast to our case, in which we noted a 2.6 decrease in α-L-fucosidase, the female described by Williamson [9] exhibited no α-L-fucosidase activity in the leucocytes. Finally, the third splice site variant, in which the c.1261-1G>A substitution affected the invariant AG acceptor splice site of intron 7, was documented in an eight-year-old Indian boy, whose parents were related [1,10]. Similarly to the our case, the boy exhibited gradual loss of motor and speech skills, leg spasticity, equinovarus deformity of the feet, short stature and facial dysmorphism, including wide mouth and thick lips [1,10]. Also, the α-fucosidase activity was low. Both genetic analysis of *FUCA1* mutations and a positive qualitative urine test based on thin layer chromatography confirmed a diagnosis of fucosidosis [1,10], as also in our case.

Nevertheless, not only splice site mutations but also mutations within the *FUCA1* sequence can cause similar clinical manifestations. For example, several symptoms resembling the clinical phenotype of our case have been reported in an eight-year-old Chinese boy with homozygous mutation within exon 2 (c.393(exon2)T>A). Among the symptoms were: coarse facial features, protruding forehead, arcuated eyebrows, broad eye gap, thick lips and maculopapular rash (and suspected telangiectasia) [33].

Although there are several well-defined symptoms of fucosidosis, it is also well known that there is large inter-individual variation in their pattern and clinical consequences. Therefore, it has been postulated that other genetic, nongenetic and/or environmental factors, and not only *FUCA1* mutations (since almost all cases of fucosidosis to date were linked to *FUCA1* mutations and α-L-fucosidase deficiency), also underlie the heterogeneity of the clinical manifestations of the disease [11]. Thus, to get further insight, we performed aCGH analysis and revealed two rare chromosomal aberrations in the child’s genome that involved an Xq22.2 gain and a 15q11.1-11.2 microdeletion. Since we did not observe these chromosomal abnormalities in the girl’s parents, we concluded that they arose de novo. In this case, the gain at the Xq22.2 segment encompassed four genes: *TMSB15B* (OMIM #301011), *H2BFXP*, *LOC100101478* and *H2BFWT* (OMIM #300507). To date, there is only one report documenting a gain at Xq22.2 that contained three of the abovementioned genes, namely *TMSB15B*, *H2BFXP* and *H2BFWT* [34]. Since this chromosomal gain (which also involved *H2BFM*) was, according to the evidence available so far, clearly linked to oligozoospermia [34], it certainly was not associated with the symptoms that were exhibited by the girl in this study.

Also, the 15q11.1-q11.2 microdeletion encompassing several genes, including two OMIM genes, namely *NBEAP1* (OMIM # 601889) and *POTEB* (OMIM # 608912), has not been linked, so far, to fucosidosis. Instead, a mosaic 15q11.1-q11.2 deletion encompassing *NBEAP1* and *POTEB* has been suggested to serve as a factor underlying pathogenesis of diffuse lymphangiomatosis with several typical symptoms, including large cystic lymphangioma over the left abdomen, thigh and vulva [35]. However, in our case, the child did not exhibit similar symptoms. On the other hand, a putative link between the observed symptoms of fucosidosis might be provided by the *miR-5701* microdeletion within the 15q11.1-11.2 segment, which we documented here. Since *miR-5701* has recently emerged as a crucial player in lysosome biogenesis [36], it may, at least indirectly, affect the lysosome function and thus underlie pathogenesis of different LSDs, including fucosidosis.

The CNVs that we found in both the mother’s and the daughter’s genomes might also be responsible for the girl’s chromosomal instability. For example, *H2BFXP*, which was involved in an Xq22.2 gain in the girl’s genome, might be one of the candidate genes engaged in chromosomal aberrations. Although *H2BFWT* is expressed exclusively in the sperm nuclei [37], where it facilitates the replacement of somatic histones by protamines during the final stages of spermatogenesis [38,39], one can assume that if CNVs affecting this gene arose de novo during the father’s spermatogenesis, it indeed might have influenced the development of the father’s primordial germ cells. If so, it might subsequently serve as an additional factor affecting fetus development. Taking into account that the aCGH analyses were conducted with the use of the genomic DNA extracted from somatic cells (blood cells), such a scenario seems to be possible. The CNVs that affected *H2BFWT* might disturb the mitotic chromosome assembly. Indeed, it has been shown that, in contrast to the canonical histone H2B, H2BFWT (because of its highly divergent NH2 tail) exhibits impairment in the recruitment of the chromosome condensation factors and does not contribute to mitotic chromosome assembly [37].

The other two genes that were affected by CNVs and might have contributed to the girl’s genome instability were involved in the mother’s 22q11.23 chromosomal gain. One of them, *SMARCB1*, has been identified as a crucial guardian engaged in the protection of CpG islands (CGIs) against de novo DNA methylation during oogenesis [40]. Moreover, SMARCB1 has been recently shown to modulate nucleosome positioning and silencing of *OCT4* (octamer-binding transcription factor 4) [29], which in turn is necessary for maintaining both the pluripotency and viability of mammalian PGCs [27].

Another putative factor that may have affected genome stability during oogenesis is PIWIL3, which is engaged in RNA-mediated silencing of transposable elements. Recently, PIWL3 has been reported as a critical player involved in genome integrity maintenance, especially in germ cells. PIWIL3’s role has also been accentuated as important during oocyte meiosis and in embryo development [30].

In general, the heterogeneity of clinical manifestations is most often related to defects in the epigenetic machinery, namely DNA methylation, a well-known contributor to the broadening of the phenotype [12]. For example, it is believed that promoter hypermethylation may lead to gene silencing and thus mimic loss-of-function mutations and/or microdeletions involving the particular gene/s [32]. Indeed, a genome-wide analysis of the girl’s blood cell methylome revealed changes in its pattern that involved a wide range of the metabolic-related and biochemical pathways, as well as highlighting the role of DNA methylation in the pathogenesis of the disease and its clinical course. For example, from amongst the group of genes with aberrant methylation patterns that might have contributed to developmental delay and psychomotor regression, including cognitive and speech impairment, hypertonia and progressive spasticity of the girl’s body, we can list *DNM2* (dynamin 2) (infection and inflammation pathways—Figure 5, OMIM # 602378) [41], *SLC2A1/GLUT-1* (Solute Carrier Family 2 Member 1) (infection and inflammation pathways, phosphoprotein, carbohydrate-insulin pathways, lipid metabolism pathways—Figure 5, OMIM # 606777), *ATP1A3* (ATPase Na+/K+ Transporting Subunit α 3) (carbohydrate-insulin pathways, lipid metabolism pathways—Figure 5, OMIM # 182350), *CACNA1A* (Calcium Voltage-Gated Channel Subunit Alpha1 A) (calcium-mediated pathways, carbohydrate-insulin pathways—Figure 5, OMIM # 601011) and *KCNMA1* (Potassium Calcium-Activated Channel Subfamily M α 1) (calcium-mediated pathways, carbohydrate-insulin pathways—Figure 5, OMIM # 600150) [42,43,44,45]. Furthermore, loss-of-function mutations of *CACNA1A* have been clearly linked to benign paroxysmal torticollis of infancy [46] as well as to focal dystonia [47]. In turn, with regard to the muscle malfunction, we noted that numerous DMS were enriched in the vascular smooth muscle contraction pathway, in which the crucial role is played by KCNMA1 [48].

In conclusion, although the newly identified *FUCA1* mutation is probably the main contributor to the pathogenesis and clinical consequences of fucosidosis reported in this study, additional factors, such as two chromosomal aberrations and changes in blood cell methylome, might expand the spectrum of observed symptoms.

## Figures and Tables

**Table 1:** PCR amplification conditions.

*FUCA1*	Genomic Position [bp] of PCR Amplicon	PCR Length [bp]	Ta [°C]	5′-3′ Forward	5′-3′ Reverse
Exon 1	Chr1:23867835-23868368	533	61	AGCCCCACCTCCTGTTTG	GGGCCAATCGTTAGTCAGAG
Exon 2	Chr1:23865244-23865980	736	60	CTGTAACGGTGTCCCCTTTG	CTCCTTCAGGGCAAAGAATG
Exon 3	Chr1:23862935-23863410	475	61	GCAATATGGCCACATGACAG	GGTTTTGGATCCCATTCCTC
Exon 4	Chr1:23859621-23860301	680	59	AGCCAGGGAAGATTAAAGGAG	TGCTGTGTTTGTTTTAAATGGTG
Exon 5	Chr1:23854026-23854815	789	TDPCR	TCCCCCTCTGTGAGAAACAC	GCTGTCCTGTGCATTGTAGG
Exon 6	Chr1:23848438-23848888	450	60	TCACCAAACCCACTCTCTCC	CCAGGCTTGGCATTCATAG
Exon 7	Chr1:23845925-23846344	419	59	GGTAGGAAACAGCCACGC	AGCCAACATCACACCATTGC
Exon 8	Chr1:23845237-23846005	768	61	CAGCCTGGTAAGCCTTTTCC	CTTCCCTGCCAGGTTTCTC
Exon 8	Chr1:23844873-23845347	474	59	TCTAACAAAGAGGCTGAACTGG	AGGATTTGGCAAGCTCAACC

**Table 2:** The list of genes subjected to copy number variations (CNVs) resulting from the mother’s 22q11.23 chromosomal gain, the daughter’s 15q11.1-q11.2 microdeletion and the daughter’s X.22.2 chromosomal gain.

Mother’s 22q11.23 Gain	Daughter’s 15q11.1-q11.2 Microdeletion	Daughter’s X.22.2 Gain
*BCR* (Breakpoint Cluster Region)	*HERC2P3* (Hect Domain And RLD 2 Pseudogene 3)	*TMSB15B* (Thymosin β 15B)
*CES5AP1* (Carboxylesterase 5A Pseudogene 1)	*GOLGA6L6* (Golgin A6 Family Like 6)	*H2BFXP* (H2B.W Histone 4, Pseudogene)
*ZDHHC8P1* (Zinc Finger DHHC-Type Containing 8 Pseudogene 1)	*GOLGA8CP* (Golgin A8 Family Member C, Pseudogene)	*LOC100101478*
*LOC101929374*	*NBEAP1* (Neurobeachin Pseudogene 1)	*H2BFWT* (H2B Histone Family Member W, Testis Specific)
*LOC388882*	*MIR3118-4* (MicroRNA 3118-4)	
*IGLL1* (Immunoglobulin Lambda Like Polypeptide 1)	*MIR3118-3* (MicroRNA 3118-3)	
*DRICH1* (Aspartate Rich 1)	*MIR3118-2* (MicroRNA 3118-2)	
*GUSBP11* (Glucuronidase, β/Immunoglobulin Lambda-Like Polypeptide 1 Pseudogene)	*POTEB3* (POTE Ankyrin Domain Family Member B3)	
*RGL4* (Ral Guanine Nucleotide Dissociation Stimulator Like 4)	*POTEB* (POTE Ankyrin Domain Family Member B)	
*ZNF70* (Zinc Finger Protein 70)	*POTEB2* (POTE Ankyrin Domain Family Member B2)	
*VPREB3* (V-Set Pre-B Cell Surrogate Light Chain 3)	*NF1P2* (Neurofibromin 1 Pseudogene 2)	
*C22orf15* (Chromosome 22 Open Reading Frame 15)	*MIR5701-3* (MicroRNA 5701-3)	
*CHCHD10* (Coiled-Coil-Helix-Coiled-Coil-Helix Domain Containing 10)	*MIR5701-1* (MicroRNA 5701-1)	
*MMP11* (Matrix Metallopeptidase 11) *SMARCB1* (SWI/SNF Related, Matrix Associated, Actin Dependent Regulator Of Chromatin, Subfamily B, Member 1)	*MIR5701-2* (MicroRNA 5701-2)	
*DERL3* (Derlin 3)	*LINC01193* (Long Intergenic Non-Protein Coding RNA 1193)	
*SLC2A11* (Solute Carrier Family 2 Member 11)	*LOC646214* (P21 (RAC1) Activated Kinase 2 Pseudogene)	
*MIF-AS1* (MIF Antisense RNA 1)	*CXADRP2* (Coxsackie Virus and Adenovirus Receptor Pseudogene 2)	
*MIF* (Macrophage Migration Inhibitory Factor)		
*GSTT2B* (Glutathione S-Transferase Theta 2B)		
*GSTT2* (Glutathione S-Transferase Theta 2, Gene/Pseudogene)		
*DDTL* (D-Dopachrome Tautomerase Like) *DDT* (D-Dopachrome Tautomerase)		
*GSTTP1* (Glutathione S-Transferase Theta 4)		
*LOC391322* (D-Dopachrome Tautomerase-Like)		
*GSTT1-AS1* (GSTT1 Antisense RNA 1)		
*GSTT1* (Glutathione S-Transferase Theta 1)		
*GSTTP2* (Glutathione S-Transferase Theta Pseudogene 2)		
*CABIN1* (Calcineurin Binding Protein 1)		
*SUSD2* (Sushi Domain Containing 2)		
*GGT5* (γ-Glutamyltransferase 5)		
*POM121L9P* (POM121 Transmembrane Nucleoporin Like 9, Pseudogene)		
*SPECC1L* (Sperm Antigen With Calponin Homology And Coiled-Coil Domains 1 Like)		
*SPECC1L* (Sperm Antigen With Calponin Homology And Coiled-Coil Domains 1 Like)		
*ADORA2A* (Adenosine A2a Receptor)		
*ADORA2A-AS1* (ADORA2A Antisense RNA 1)		
*UPB1* (β-Ureidopropionase 1)		
*GUCD1* (Guanylyl Cyclase Domain Containing 1)		
*SNRPD3* (Small Nuclear Ribonucleoprotein D3 Polypeptide)		
*GGT1* (γ-Glutamyltransferase 1)		
*LRRC75B* (Leucine Rich Repeat Containing 75B)		
*BCRP3* (Breakpoint Cluster Region Pseudogene 3)		
*POM121L10P* (POM121 Transmembrane Nucleoporin Like 10, Pseudogene)		
*PIWIL3* (Piwi Like RNA-Mediated Gene Silencing 3)		

**Table 3:** Alignment efficiency of reduced representation bisulfite sequencing (RRBS) reads against the human genome (GRCh38 version). Percentages of the aligned reads are in brackets.

RRBS Library	Total Reads (After Filtering)	Aligned Reads	Uniquely Aligned Reads
Father	16,442,608	13,169,725 (80.1%)	9,822,103 (59.7%)
Mother	18,778,091	15,678,863 (83.5%)	11,785,228 (62.8%)
Daughter	24,380,417	19,220,521 (78.8%)	14,438,686 (59.2%)

**Table 4:** The pairwise comparisons of the RRBS datasets and DMS methylation trends in the whole genomes and within the selected chromosome regions (explained in the text). Abbreviations: F—father, M—mother, D—daughter.

Type of Pairwise Comparison	Number of DMS			
	Chromosome 15	Chromosome 22	Chromosome X	All Chromosomes
Hypomethylated DMS				
F vs. D	245 (11.78%)	871 (41.93%)	25 (1.20%)	2077
M vs. D	318 (16.13%)	1042 (52.87%)	34 (1.72%)	1971
Hypermethylated DMS				
F vs. D	168 (5.58%)	896 (28.85%)	26 (0.86%)	3012
M vs. D	268 (8.40%)	1134 (35.55%)	47 (1.47%)	3190

**Table 5:** The list of molecular and biochemical pathways relevant for DMS-associated genes, separated for hypomethylated and hypermethylated DMS.

Hypomethylated DMS	Hypermethylated DMS
**Metabolic processes**	
Metabolic pathways	Metabolic pathways
Adipocytokine signaling	-
Sphingolipid signaling	Sphingolipid signaling
Propanoate metabolism	-
Nonalcoholic fatty liver disease (NAFLD)	-
Neutrophin signaling	-
Central carbon metabolism in cancer	Central carbon metabolism in cancer
Carbon metabolism	-
Carbohydrate digestion and absorption	-
-	Choline metabolism in cancer
**Cellular signaling pathways**	
Pathways in cancer	-
cAMP signaling pathway	-
-	Rap1 signaling
-	PI3K-Akt signaling
-	MAPK signaling, calcium signaling
**Biological processes**	
Regulation of actin cytoskeleton,	Regulation of actin cytoskeleton
endocytosis	-
-	Proteoglycans in cancer
-	Focal adhesion
-	Adherens junction
**Neurological processes**	
Alzheimer’s disease	-
Cholinergic synapse	Cholinergic synapse
-	Morphine addiction
-	Serotonergic synapse
-	Hippo signaling
-	Glutamatergic signaling
-	Axon guidance
-	Long-term potentiation
-	Long-term depression
**Cancerogenic processes**	
Small cell lung cancer	-
-	Melanoma
-	Non-small cell lung cancer
-	Glioma
-	Acute myeloid leukemia
Chronic myeloid leukemia	-
**Physiological processes**	
Renal cell carcinoma	Renal cell carcinoma
-	Vascular smooth muscle contraction
-	Progesterone-mediated oocyte maturation
-	Oocyte meiosis
**Cell death and autophagy processes**	
Apoptosis	Apoptosis
-	Regulation of autophagy
-	Phagosome
**Developmental processes**	
Osteoclast differentiation	-
Type II diabetes mellitus	-
-	Signaling pathways regulating pluripotency of stem cells
-	Osteoclast differentiation
**RNA metabolism**	
RNA degradation	Spliceosome
-	MicroRNA in cancers
**DNA repair processes**	
-	Nucleotide excision repair and mismatch repair

## Data Availability

The data presented in this study are available in the supplementary material. Additionally, the detected deletion was deposited in the ClinVar database under accession number SCV001450462 and RRBS sequencing reads are available in the SRA database under BioProject PRJNA655811.

## Supplementary Materials

Supplementary materials can be found at https://www.mdpi.com/2073-4425/12/1/74/s1. Figure S1. Pearson correlation coefficient of RRBS methylome data. Figure S2. The distribution of the DMS in the investigated genomes. Table S1. Hematological parameters and biochemical characteristics of the child. Table S2. A total of 529 SNP genotypes of the family trio derived from the RRBS dataset. Table S3. Comparative set of DMS sites with their genomic location and the value of methylation difference. Table S4. Annotation data for comparative set of DMS sites. Table S5. List of common imprinted genes found in the annotated DMS set. Table S6. Functional annotation chart (DAVID results with false discovery rate below 0.05). Table S7. List of genes encoding phosphoproteins. Table S8. List of genes overrepresented in chromosomal rearrangement. Table S9. List of genes overrepresented in mental retardation.
