# Supplementary material for: The Identification of a Novel Fucosidosis-Associated FUCA1 Mutation: A Case of a 5-Year-Old Polish Girl with Two Additional Rare Chromosomal Aberrations and Affected DNA Methylation Patterns
Source: Genes (Basel). 2021 Jan 8;12(1):74. doi: 10.3390/genes12010074 (PMC7827884; doi:10.3390/genes12010074)
Supplement: Supplementary file 1 [file genes-12-00074-s001.zip › supplementary/Supplementary figures.doc]

Figure S1

Figure S1. Pearson correlation coefficient of RRBS methylome data.

Figure S2

**Figure S2.** The distribution of the DMS in the investigated genomes. Abbreviations: **3 prime UTR variant** - a CpG sequence variant of the untranslated region of the 3' end of a gene; **5 prime UTR variant** - a CpG sequence variant of the untranslated region of the 5' end of a gene; **downstream gene variant** - a CpG sequence variant located downstream to the 3' end of a gene; **intergenic variant** - a sequence CpG variant located in the intergenic region, between genes; **intron variant** - a CpG sequence variant occurring within an intron of a gene; **mature miRNA variant** - a transcript CpG variant located in the sequence of the mature miRNA; **missense variant** - a CpG sequence variant, that changes one or more bases, resulting in a different amino acid sequence; **NMD transcript variant** - a CpG variant of the transcript sequence that is the target of mRNA genes from the nonsense-mediated decay pathway; **non coding transcript exon variant** - a CpG variant of the exonic sequence in a non-coding transcript; **non coding transcript variant** - a CpG transcript variant of a non-coding RNA gene; **regulatory region variant -** a CpG sequence variant located within a regulatory region**.**
